# Direct metagenomic and amplicon-based Nanopore sequencing of French human monkeypox from clinical specimen

**DOI:** 10.1128/MRA.00811-23

**Published:** 2023-12-04

**Authors:** Aurelia Kwasiborski, Véronique Hourdel, Charlotte Balière, Damien Hoinard, Quentin Grassin, Maxence Feher, Clémentine De La Porte Des Vaux, Mélanie Cresta, Jessica Vanhomwegen, Jean-Claude Manuguerra, Christophe Batéjat, Valérie Caro

**Affiliations:** 1 Institut Pasteur, Université Paris Cité, Environment and Infectious Risks Unit, Laboratory for Urgent Response to Biological Threats, Paris, France; 2 Bichat-Claude Bernard Hospital, Paris, France; Katholieke Universiteit Leuven, Leuven, Belgium

**Keywords:** monkeypox virus, metagenomics, amplicon-based sequencing, nanopore, next-generation sequencing

## Abstract

We report the whole-genome sequence of monkeypox virus obtained using MinION technology (Oxford Nanopore Technologies) from a French clinical specimen during the 2022 epidemic. Amplicon-based sequencing and shotgun metagenomic approaches were directly applied to the sample.

## ANNOUNCEMENT

Monkeypox virus (MPXV), a double-stranded DNA virus with a genome of approximately 200 kb and belonging to the genus *Orthopoxvirus* of the family *Poxviridae*, is the causative agent of monkeypox recently renamed mpox (1). In 2022, this zoonotic disease was responsible for the most widespread and largest outbreak outside Africa (2). This global outbreak has strengthened the need for rapid genomic-based surveillance tools. Here, we report the genomic sequence of MPXV based on a comparison between direct metagenomic sequencing as already referenced (3, 4) and an amplicon-based approach using the MinION.

An oropharyngeal swab was collected from a 35-year-old French man who consulted Bichat-Claude Bernard Hospital, Paris France, in June 2022, for mpox suggestive lesions and was sent to the Laboratory for Urgent Response to Biological Threats for diagnostic testing (specimen identification number CIBU-220031).

Genomic DNA was extracted using the QIAamp DNA blood kit (QIAGEN) according to the manufacturer’s recommendations. The clinical specimen was found positive with a threshold cycle value of 23 using an MPXV real-time PCR assay targeting the *G2RG* gene (5).

For the metagenomics approach (CIBU-220031-M), a DNA library was prepared from 1 ng of DNA using the Rapid PCR Barcoding kit (SQK-RPB004) and loaded onto an R9.4.1 flow cell for 24 hours of sequencing run on the Mk1C device. The nextflow workflow (wf-mpx version 0.0.4) was applied to FastQ files with medaka (1.5.0) for mapping to the reference MT903344, flye (2.9-b1768) for assembly and medaka (1.5.0), bedtools (v2.30.0), and bcftools (1.15) for consensus generation (6, 7).

For the amplicon-based method (CIBU-220031-A), a multiplex MPXV PCR protocol was applied to genomic DNA (8). Obtained PCR products were purified using a 1:1 ratio of Ampure XP clean up beads (Beckman Coulter). The library was prepared using Ligation Sequencing (SQK-LSK109) and Native Barcoding kits (EXP-NBD104/114) and loaded onto R9.4.1 flow-cell for 24 hours on the Mk1C. The field bioinformatics protocol proposed by the ARTIC Network was adapted for data analysis and consensus sequence generation. Guppy (v6.0.7), minimap2 (v2.17-r941) (9), and medaka (v1.0.3) were, respectively, used for basecalling, de-multiplexing, mapping, and consensus generation. Cramino (v0.9.7) and Tablet (v1.21.02.08) (10) were used to calculate the number of reads and the percentage of genome coverage and depth. Default parameters were applied.

The runs respectively generated 4.27 and 3.33 million reads for CIBU-220031-M and CIBU-220031-A (Table 1). Mapping against the reference MT903344 showed 99.97% and 99.42% genome coverage with an average depth of 462× and 577×, respectively. Consensus sequences of 197,233 bp for CIBU-220031-M and 197,179 bp for CIBU-220031-A were generated. The length difference is due to the failure to obtain the genomic ends with the amplicon-based method. Both viral genome sequences showed 100% nucleotide identity between them and 99.2% nucleotide identity with the reference MT903344, unveiling 47 nucleotide substitutions (5 out of CDS, 19 synonymous, and 23 non-synonymous). The viral sequence was lineage B.1. in clade IIb according to the Nextclade application (11).

**TABLE 1:** Nanopore sequencing data for sample CIBU-220031-M and CIBU-220031-A

Feature	CIBU-220031-M	CIBU-220031-A
No. of total reads (M)	4.27	3.33
No. of mapped reads (M)	34,760	59,794
Mean length of reads (bp)	2,868	2,073
Mean quality score	11.6	12
Average depth (×)	462	577
Maximum coverage depth	733	800
Consensus length (bp)	197,233	197,179
GC content (%)	33	33

Both approaches have proved successful in obtaining correct genomic sequences. However, the amplicon-based approach yields more targeted reads, increasing the depth coverage. Thus, this method would enable more samples to be sequenced in a single MinION run, reducing the cost and time, which can be important factors when dealing with an infectious disease.

## Data Availability

The consensus genome sequence was deposited in the GISAID EpiPox database (accession number EPI_ISL_18128768) and GenBank (accession number OR473631). The raw reads were deposited in the NCBI Sequence Read Archive under accession number PRJNA1004259.
